# Two different troponin isoforms for detecting early myocardial injury after curative resection of oesophageal cancer

**DOI:** 10.1186/s13019-020-01225-9

**Published:** 2020-07-25

**Authors:** Wei Gu, Wei Tang, Zuojing Zhang, Meiying Xu, Jingxiang Wu

**Affiliations:** Department of Anaesthesiology, Shanghai Chest Hospital, Shanghai Jiao Tong University, No. 241 Huaihai Rd. West, Shanghai, China

**Keywords:** Anaesthesia, Postoperative myocardial, Oesophageal Cancer, Troponin

## Abstract

**Background:**

The objective of this study was to explore the consistency and correlation of two troponin (cTn) subtypes, troponin I (cTnI) and high-sensitivity troponin T (hs-cTnT), which can be used to judge early myocardial injury after curative resection of oesophageal cancer.

**Methods:**

This study is a secondary analysis of data obtained from a previous randomized controlled trial on postoperative myocardial injury in 70 patients undergoing elective curative resection of oesophageal cancer who were randomly assigned to undergo aggressive body temperature management (nasopharyngeal temperature 36.61 ± 0.18 °C) or standard body temperature management (35.80 ± 0.18 °C, *n* = 35 in each arm). The serum cTnI and hs-cTnT levels were measured in each patient at the 4 time points: before the operation and 6 h ~ 12 h, 24 h and 48 h after the operation. The diagnostic criteria of myocardial injury followed the third edition ESC/ACCF definition of myocardial infarction. The primary outcomes included the following: (1) the incidence of myocardial injury and the relationship between hs-cTnT and cTn and (2) the consistency and correlation of the two cTn subtypes.

**Results:**

A total of 280 pairs of cTn samples were tested. The incidence of postoperative day 2 myocardial injury was 8.6% (3/35) among patients receiving aggressive body temperature management and 31.4% (11/35) among patients receiving standard body temperature management (*P <* 0.05). Among 3 patients who experienced myocardial injury in the aggressive body temperature management group, 2 met the diagnostic criteria for cTnI and hs-cTnT and only 1 met the diagnostic criteria for hs-cTnT. Among the 11 patients who experienced myocardial injury in the standard body temperature management group, 7 met the diagnostic criteria for cTnI and hs-cTnT and only 3 met the diagnostic criteria for hs-cTnT; only 1 met the diagnostic criteria for cTnI. The bias of cTnI and hs-cTnT was − 8.82 ± 31.91 ng/L. The consistency limit was − 71.37 ~ 53.73 ng/L. The proportion within the scope of the consistency of its corresponding boundary was 98.57%. The correlation coefficient of cTnI and hs-cTnT was 0.845 (*P <* 0.05).

**Conclusions:**

In the evaluation of postoperative myocardial injury in patients undergoing curative resection of oesophageal cancer, cTnI and hs-cTnT exhibit high consistency and a good correlation. The combination of cTnI and hs-cTnT can improve the detection rate of myocardial injury, thus providing a better reference than a single measure alone for reducing the risk of perioperative myocardial injury in patients.

**Trial registration:**

ChiCTR-INR-17011621. Registered June 10, 2017.

## Background

The incidence of myocardial injury after major non-cardiac surgery is approximately 8%, and myocardial injury is one of the major risk factors for perioperative adverse events [1]. Currently, high-sensitivity troponin T (hs-cTnT) and cTnI are commonly used in clinical practice to diagnose myocardial injury and predict cardiac complications after non-cardiac surgery. However, the better option between cTnI and cTnT remains a question of debate. This study is a secondary analysis of data obtained from a previous randomized controlled trial on postoperative myocardial injury in middle-aged and elderly patients following curative resection of oesophageal cancer with aggressive or standard body temperature management (registered at http://www.chictr.org.cn/showproj.aspx? Proj =18,675; the results have been published in the journal *Anesthesia & Analgesia* [2]). In this study, hs-cTnT and cTnI were compared to reflect the changes in the myocardial injury detection rate. Curative resection of oesophageal cancer as a major non-cardiac operation is characterized by a wide range of operations, a long operation time, and large trauma. We found that patients undergoing curative resection of oesophageal cancer are prone to perioperative hypothermia and are at a relatively high risk of postoperative myocardial injury. Myocardial injury most often occurs within postoperative day 2. Among the patients who experience myocardial injury, 16% exhibit symptoms of myocardial ischaemia and 84% exhibit no symptoms of myocardial ischaemia and no significant electrocardiogram changes; therefore, myocardial injury can be diagnosed only by elevated troponin (cTn) [3]. The European Society of Cardiology (ESC)/American College of Cardiology Foundation (ACCF)/American Heart Association (AHA)/World Heart Federation (WHF) jointly issued the third edition definition of myocardial infarction. According to the definition, slight myocardial injury can be detected by cTn in the early postoperative period [4]. cTn exists only in cardiomyocytes and is a complex composed of troponin T (cTnT), troponin I (cTnI) and troponin C (cTnC) [5]. hs-cTnT is a unique regulatory protein located on cardiac fibres. The minimum concentration of the fifth-generation hs-ctnt STAT assay is 3 pg/ml, and its positive predictive value is approximately 95%. cTnI is a unique contractility protein, its normal values range from 0 to 0.03 g/L, cTnI ≥0.04 g/L can be diagnosed as myocardial infarction [6], and the positive predictive value of the third-generation enhanced AccuTnI assay is approximately 60%. Although hs-cTnT and cTnI are both sensitive indicators for the detection of myocardial injury, hs-cTnT and cTnI have the risk of missing early myocardial injury. The purpose of this study was to explore the value of hs-cTnT and cTnI in the determination of early myocardial injury after curative resection of oesophageal cancer to improve the detection rate of early postoperative myocardial injury and reduce the risk of perioperative myocardial injury in patients.

## Methods

### Patients

This study is a secondary analysis of data obtained from a previous randomized controlled trial on postoperative myocardial injury in middle-aged and elderly patients following curative resection of oesophageal cancer with aggressive or standard body temperature management [2]. This study protocol was approved by the Ethics Committee of Shanghai Chest Hospital (Institutional Review Board# KS1614), and written informed consent was obtained from all subjects participating in the trial before enrolment. The trial was registered before patient enrolment at chictr.org.cn (ChiCTR-INR-17011621, principal investigator: Jingxiang Wu, date of registration: June 10, 2017).

Seventy-five patients scheduled for elective oesophageal cancer surgery were assessed for eligibility. All patients underwent thoracoscopic triple incision oesophageal carcinoma surgery (McKeown surgery). Patients underwent an open oesophagectomy in the left lateral decubitus position and were then switched to the supine position with the head tilted to the right to complete the abdominal and neck surgery. The inclusion criteria were as follows: (1) patients classified as having an ASA physical status of I or II and who had been scheduled to receive curative resection of oesophageal carcinoma under general anaesthesia, (2) age 45–80 years, and (3) expected surgery duration of 2–6 h. The exclusion criteria were as follows: (1) major cardiovascular diseases at baseline; (2) preoperative serum cTnI > 0.03 ng/mL; (3) body mass index > 30 kg/m^2^; (4) end-stage renal disease requiring dialysis; (5) major infection, including but not limited to septicaemia; or (6) clinically important coagulopathy.

### Measurements

Based on a computer-generated randomization sequence, patients were randomly assigned to receive either aggressive or standard body temperature management. Patients were randomly assigned to each group at a 1:1 ratio. An opaque, sealed envelope was opened to determine the patient’s group assignment after the patient had provided written informed consent. Patients were not informed of their treatment assignment. In the standard body temperature management arm, the heating pad was off on patient arrival and turned on only if the body temperature dropped to 35.0 °C. An additional heating pad was applied to the neck and shoulder areas if the body temperature remained at < 35.0 °C for 20 min. The heating pad was turned off if the body temperature reached 35.2 °C. In the aggressive management arm, the heating pad was turned on upon patient arrival and turned off if body temperature increased to 37.0 °C. It was turned on if the body temperature decreased to 36.5 °C. An additional heating pad was applied to the neck and shoulder areas if the body temperature dropped to 36.0 °C. The same surgeons operated on both groups and were not blinded to allocation.

Blood was drawn before surgery and at 6–12, 24, and 48 h after surgery. A secondary analysis was performed on the abovementioned frozen blood samples for the measurement of serum hs-cTnT and cTnI. cTnI levels were assayed using a third-generation enhanced AccuTnI assay (Beckman Coulter, Brea, CA), while hs-cTnT levels were assayed using a fifth-generation hs-cTnT STAT assay (Roche Diagnostics, Shanghai, China). The laboratory technicians performing these assays were blinded to patient allocation.

Myocardial injury was defined as cTnI > 0.06 μg/L during the first 2 postoperative days; this was the lowest measurable value, with a 10% coefficient of variation above the 99th percentile of 0.04 μg/L for the assay used [7]. Alternatively, myocardial injury was defined as the occurrence of hs-cTnT ≥0.065 μg/L or 0.02 μg/L ≤ hs-cTnT< 0.065 μg/L but with an absolute change of at least 0.005 μg/L. [1, 4] Myocardial injury was defined as elevated cTnI or hs-cTnT because the 2 measures carry distinct sensitivity and specificity and are both implicated in myocardial injury. Additionally, the timing of the 2 measures was different.

### Statistical analysis

SPSS 22.0 (IBM, Chicago, IL) was used for data analysis. Data were tested for normal distribution, and data showing a normal distribution are expressed as the mean ± standard deviation (SD). Data showing a skewed distribution are expressed as the median (interquartile range). Chi-square tests were used for categorical variables based on the two cTn concentrations. The incidence of myocardial injury was calculated. A Venn diagram was drawn for the diagnosis of myocardial injury with hs-cTnT and cTnI elevation. The consistency between the two cTn concentrations was analysed by the Bland-Altman method. Bias is defined as the mean of the difference between two cTn concentrations and represents systematic error, whereas SD of bias represents random error or variability; the consistency limit is bias±2 SD. The percentage of the difference between the two cTn concentrations within the consistency threshold indicates the percentage within the consistency limit. Pearson’s correlation analysis was performed to compare the two cTn concentrations. *P*-values < 0.05 were considered statistically significant.

## Results

### Baseline characteristics

**Fig. 1:**
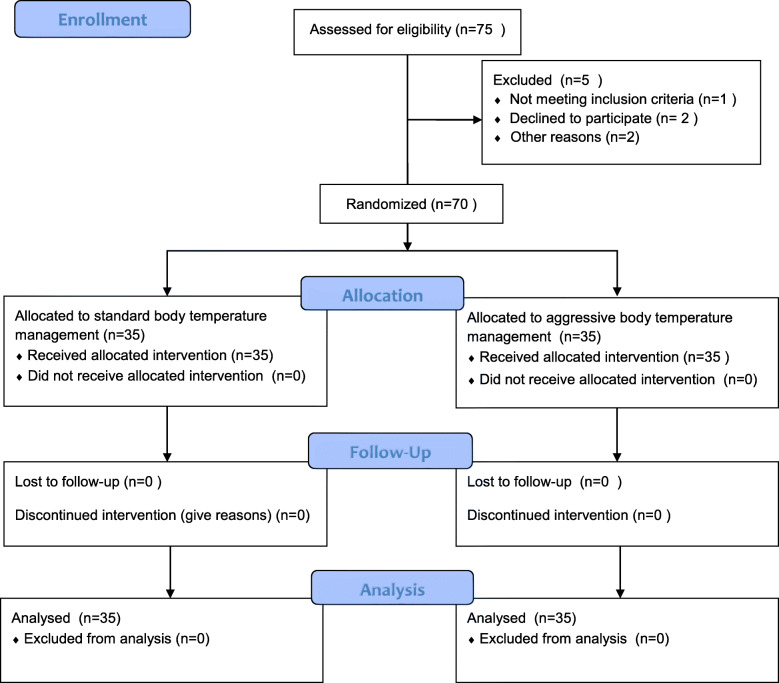
Flowchart of patients enrolled in the study

We initially assessed 75 patients for eligibility, of whom 70 were enrolled, and all completed follow-up (Fig. 1). A total of 280 test data sets were collected, including 210 test data sets 48 h after surgery. According to 210 test data sets 48 h after surgery, 3 patients in the aggressive body temperature management group experienced myocardial injury, and 11 patients in the standard body temperature management group experienced myocardial injury. T tests were performed in both groups. According to the incidence of myocardial injury, a Venn diagram and a scatter diagram of the relationship between cTnI and hs-cTnT were drawn. A total of 140 data pairs were included in the Bland-Altman consistency analysis and Pearson’s correlation analysis.

### Diagnostic efficacy for myocardial injury

**Fig. 2:**
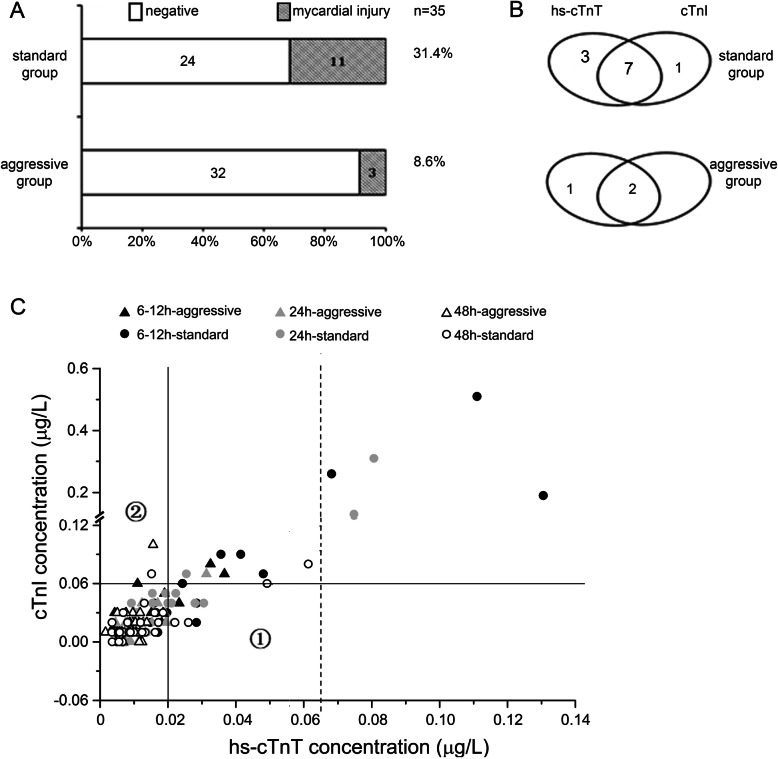
The effect of two diagnostic criteria for cTn on detecting the incidence of myocardial injury after oesophageal cancer surgery with aggressive or standard body temperature management. **a** Incidence of myocardial injury after curative resection of oesophageal cancer. **b** Venn diagram of the two diagnostic criteria for cTn. **c** Scatter plots of cTnI and hs-cTnT (210 pairs of samples at postoperative 6 h ~ 12 h, 24 h and 48 h). The data above the horizontal line conform to the diagnostic criteria for cTnI. The data to the right side of the vertical line conform to the diagnostic criteria for hs-cTnT (the dotted vertical line represents hs-cTnT ≥0.065 g/L, and the solid vertical line represents 0.02 g/L ≤ hs-cTnT < 0.065 g/L; hs-cTnT was increased by at least 0.005 g/L compared with the preoperative level). The triangles represent the aggressive body temperature management group, and the circles represent the standard body temperature management group

The incidence of myocardial injury during the first 2 postoperative days was 8.6% (3/35) in the aggressive body temperature management group and 31.4% (11/35) in the standard body temperature management group (*P* < 0.05, Fig. 2a). Among patients who experienced myocardial injury, both the cTnI and hs-cTnT criteria for myocardial injury were met in 3 patients in the aggressive body temperature management group and 11 patients in the standard body temperature management group. Based only on the cTnI criterion, myocardial injury occurred in 2 patients in the aggressive body temperature management group and 8 patients in the standard body temperature management group. Based only on the hs-cTnT criterion, myocardial injury occurred in 3 patients in the aggressive body temperature management group and 10 patients in the standard body temperature management group (Fig. 2b).

The number of myocardial injury samples and the concentration of cTn in the aggressive body temperature management group were significantly lower than those in the standard body temperature management group at postoperative 6 h ~ 12 h, 24 h and 48 h (Fig. 2c). Based only on the cTnI criterion, the area shown in ① would be missed. Based only on the hs-cTnT criterion, the area shown in ② would be missed. However, based on both the cTnI and hs-cTnT criteria, more myocardial injuries would be found to avoid a missed diagnosis and for the timely treatment of patients. Therefore, myocardial injury can more easily be detected, the number of missed diagnoses can be reduced, and patients can be treated in a timely manner.

### Bland-Altman consistency analysis

**Fig. 3:**
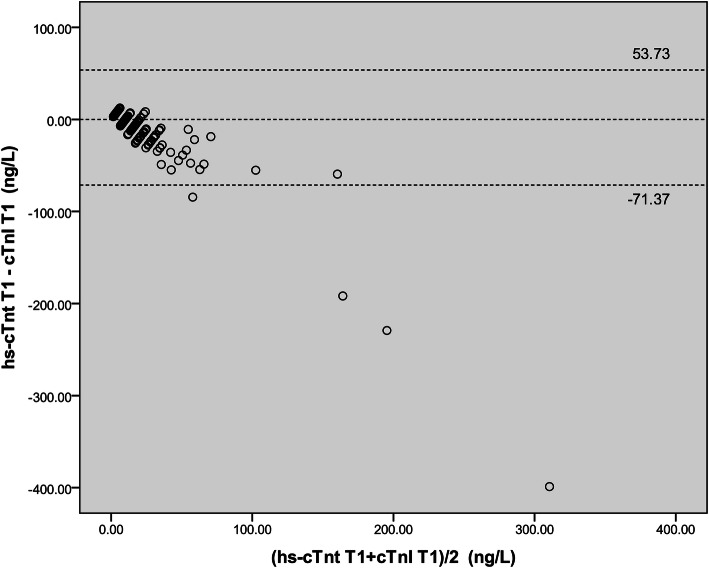
Bland-Altman scatter diagram

The scatter diagram of the difference between hs-cTnT and cTnI plotted against the average of the two shows that the bias is - 8.82 ± 31.91 ng / L and that the consistency limits are - 71.37-53.73 ng / L, indicating that the consistency between hs-cTnT and cTnI is good; the percentage within the consistency limit is 98.57% (Table 1) (Fig. 3).

**Table 1:** Comparison of bias, standard deviation and consistency limits between hs-cTnT and cTnI

Index	hs-cTnT (ng/L, $\frac{}{x}$ ±SD)	cTnI (ng/L, $\frac{}{x}$ ±SD)	Bias (ng/L)	Standard deviation of bias (ng/L)	Consistency limit		Percentage within the consistency limit (%)
					low	high	
Count	11.39 ± 13.83	20.21 ± 42.74	−8.82	31.91	−71.37	53.73	98.57%

### Pearson’s correlation analysis

**Fig. 4:**
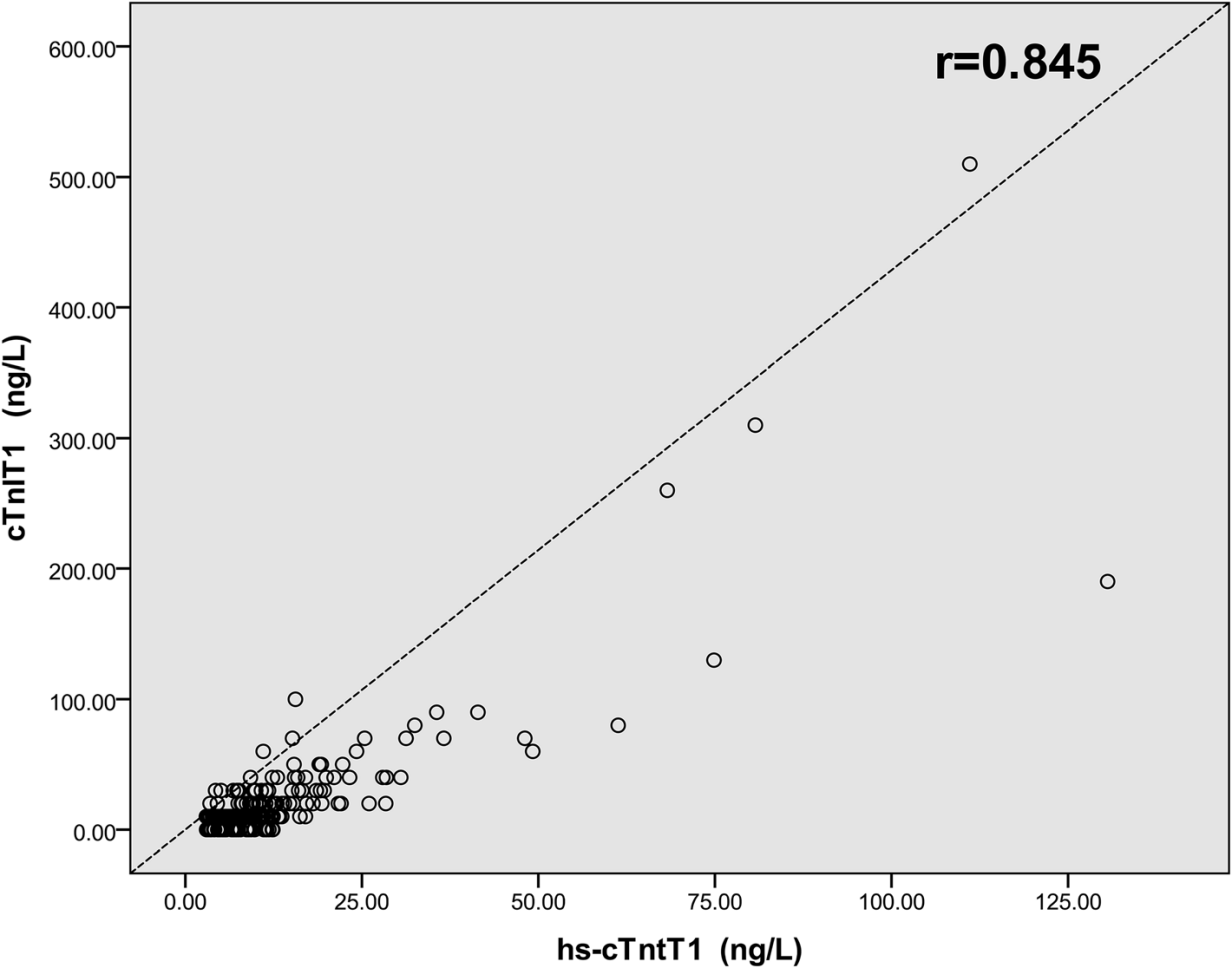
Pearson’s correlation analysis of hs-cTnT and cTnI

The correlation analysis (Pearson: r) between cTnt and cTnl is shown in Fig. 4. Good positive correlation was observed. The correlation coefficient (r) between hs-cTnT and cTnI was 0.845 (*P* < 0.01, Fig. 4).

## Discussion

The incidence of perioperative hypothermia, which is likely to cause perioperative myocardial injury, is high during curative resection of oesophageal cancer under general anaesthesia [3]. When myocardial injury occurs, cTn and creatine kinase isoenzyme (ck-mb) in the blood increase. cTn has higher sensitivity and specificity than ck-mb, which is widely used in clinical practice [8]. cTn is a new marker for the diagnosis of myocardial injury. Clinically, cTn includes cTnI and cTnT, which have high sensitivity and specificity to myocardial injury. cTnI and cTnT are currently considered the best diagnostic markers and have become the “gold standard” for the diagnosis of myocardial injury [9–11]. Therefore, the postoperative monitoring of hs-cTnT and cTnI can effectively improve the detection rate of early postoperative myocardial injury, which is particularly important in the prevention of perioperative myocardial injury.

hs-cTnT is a specific regulatory protein, and cTnI is a specific contractile protein on myocardial fibres. When the myocardial cell membrane is intact, neither hs-cTnT nor cTnI can penetrate the cell membrane into the peripheral blood circulation, so hs-cTnT and cTnI cannot be detected in the peripheral blood of healthy people [5]. cTnI, a non-enzymatic serum marker with high specificity and sensitivity, is one of the most commonly used markers for detecting myocardial injury. When the myocardium is damaged, cTnI is released into the blood, which increases at 3–5 h, peaks at 24 h, and decreases to normal at 5–10 days. hs-cTnT is highly sensitive to myocardial injury, which can appear in the early stage of myocardial injury, and can even detect the release of free cTnI and trace cTnI in the cytoplasm. When cTnI cannot be detected, hs-cTnT can be detected at a very low concentration, improving the early diagnosis of myocardial infarction and saving the lives of patients with strong timely support [12–14]. Meanwhile, when hs-cTnT cannot be detected, cTnI might be an alternative. Therefore, they can be a complementary method to improve the detection rate of myocardial injury.

This study explored the value of hs-cTnT and cTnI in the determination of early myocardial injury after curative resection of oesophageal cancer. The results showed that if hs-cTnT and cTnI were simultaneously detected within the first 2 postoperative days, the incidence of myocardial injury was 8.6% (3/35) among patients receiving aggressive body temperature management and 31.4% (11/35) among patients receiving standard body temperature management. If only cTnI was detected, the incidence of myocardial injury was 5.7% (2/35) among patients receiving aggressive body temperature management and 25.7% (9/35) among patients receiving standard body temperature management. Similarly, if only hs-cTnT was detected, the incidence of myocardial injury was 8.6% (3/35) among patients receiving aggressive body temperature management and 28.6% (10/35) among patients receiving standard body temperature management. Therefore, the combination of cTnI and hs-cTnT as the diagnostic standard of myocardial injury can be used to more easily detect myocardial injury to avoid missed diagnoses and to treat patients in a timely manner.

To clarify the correlation and reliability of cTnI and hs-cTnT, this study further analysed the consistency and correlation between cTnI and hs-cTnT. Bland-Altman consistency analysis showed that the percentage within the consistency limit of hs-cTnT and cTnI was 98.57%, which was higher than the 95% standard, indicating good consistency. The Pearson correlation coefficient of hs-cTnT and cTnI was above 0.8, indicating a good correlation. Therefore, cTnI and hs-cTnT have complementary effects in the detection of myocardial injury.

Several limitations to this study exist. First, the subjects were only patients who received curative resection of oesophageal cancer, and no other patients were studied. Second, the sample size of this study was small; only 14 patients suffered myocardial injury within the first 2 postoperative days. Finally, there is currently a lack of long-term follow-up of these patients with myocardial injury. This will require further research.

## Conclusions

In the evaluation of postoperative myocardial injury in patients undergoing curative resection of oesophageal cancer, cTnI and hs-cTnT exhibit high consistency and a good correlation. The combination of cTnI and hs-cTnT can improve the detection rate of myocardial injury, thus providing a better reference than a single measure alone for reducing the risk of perioperative myocardial injury in patients. Therefore, using both is a recommended method.

## Data Availability

The datasets used and/or analysed during the current study are available from the corresponding author on reasonable request.

## Abbreviations

cTn: troponin, troponin T (cTnT), troponin I (cTnI), and troponin C (cTnC)
hs-cTnT: high-sensitivity troponin T
ESC: European Society of Cardiology
ACCF: American College of Cardiology Foundation
AHA: American Heart Association
WHF: World Heart Federation
